# Variation in volatile and flavonoid profiles of *Cedrus libani* A. Rich. leaves along an elevational gradient

**DOI:** 10.1038/s41598-026-52703-4

**Published:** 2026-05-10

**Authors:** Fatma Merve Nacakcı

**Affiliations:** Faculty of Forestry, Isparta University of Applied Sciences, Isparta, 32260 Turkey

**Keywords:** Volatile components, Flavonoids, Terpenes, Terpenoids, PCA, Biochemistry, Plant sciences

## Abstract

This study investigates variation in the phytochemical composition of *Cedrus libani* A. Rich. leaves across an altitudinal gradient, focusing on volatile and flavonoid profiles. Leaf samples were collected from three elevations (1300, 1500, and 1700 m) in the Yukarı Gökdere Forest region (Isparta, Turkey). Volatile compounds were analyzed using GC-MS, and flavonoid compounds were determined by RP-HPLC. A total of 45 volatile compounds were identified, predominantly consisting of monoterpenes and sesquiterpenes. Major components included limonene, β-myrcene, α-pinene, caryophyllene, and germacrene-D, with significant variation observed among elevation groups. One-way ANOVA indicated that several volatile compounds differed significantly across elevations (*p* < 0.05). Flavonoid analysis showed that rutin and kaempferol decreased with increasing elevation, whereas quercetin and catechin exhibited higher values at higher elevations. All analyzed flavonoid compounds showed statistically significant differences among elevation groups (*p* < 0.05). Principal component analysis (PCA) revealed separation patterns among samples based on their chemical composition. Overall, the results indicate that the phytochemical composition of *C. libani* leaves varies along the studied elevational gradient. These findings provide a preliminary and descriptive assessment of altitude-related chemical variation and highlight the need for further studies incorporating broader sampling designs and environmental measurements.

## Introduction

The Pinaceae family represents one of the most important groups of gymnosperms (conifers), comprising 11 genera and more than 260 species widely distributed across the Northern Hemisphere, where they form a major component of mountain forest ecosystems^1^. Members of this family are known to produce a wide range of terpenoid compounds, which occur as oleoresins or essential oils and are considered valuable chemotaxonomic markers in conifer species^2,3^.

*Cedrus libani* A. Rich. is a conifer species belonging to the genus Cedrus within the Pinaceae family and is commonly known as Taurus cedar (Lebanon cedar). The species is mainly distributed in the Taurus Mountains of southern Turkey, with additional populations in the Black Sea region and neighboring countries such as Lebanon and Syria^4,5^. It typically occurs across a broad elevational range, generally between 800 and 2100 m above sea level^4^.

Forest trees are important natural sources of volatile organic compounds (VOCs), a diverse group of secondary metabolites produced by plants^6^. These compounds are mainly composed of terpenes and terpenoids and play a role in plant defense against herbivores and environmental stress factors^7–10^.

Environmental variables such as temperature, radiation, and moisture vary along altitudinal gradients and may influence the synthesis and accumulation of plant secondary metabolites^11–13^. Previous studies have reported altitude-related variation in volatile and phenolic compounds; however, the observed patterns are not consistent across species and study conditions^13–19^.

Phenolic compounds, including flavonoids, represent an important group of plant secondary metabolites involved in plant defense processes^22^. Their synthesis and accumulation may be influenced by environmental factors such as altitude. Previous studies have reported variation in phenolic composition along elevational gradients, although the observed patterns may differ depending on species and environmental conditions^23^.

Previous studies on *C. libani* and related conifer species have identified major volatile components such as α-pinene, β-pinene, limonene, β-caryophyllene, and germacrene-D, as well as flavonoid compounds including rutin, quercetin, and kaempferol^24,25^. These studies also indicate that chemical composition may vary among populations and environmental conditions^26^.

Despite these findings, information on the variation of volatile and flavonoid composition of *C. libani* leaves across different elevation levels remains limited. Although altitude is known to influence plant secondary metabolites, its effect on the chemical profiles of *C. libani* leaves has not been sufficiently characterized. Therefore, the aim of this study is to provide a descriptive assessment of variation in volatile and flavonoid profiles of *C. libani* leaves collected from three elevation levels within a single forest area.

## Results

### Content of volatile compounds

Analysis of volatile compounds in leaf samples collected from different elevations revealed the presence of 45 components (Table 1), including monoterpenes, sesquiterpenes, and terpenoids. Among these, monoterpenes and sesquiterpenes constituted the major fraction of the volatile profile. The dominant compounds included limonene, β-myrcene, and α-pinene, with their relative abundances varying across elevation levels. In addition, sesquiterpenes such as germacrene-D, caryophyllene, δ-cadinene, and γ-cadinene were detected at relatively high levels. Minor amounts of oxygenated compounds, including (E)-2-hexenal, hexanal, and (Z)-hex-3-en-1-ol, were also identified. The volatile composition was characterized by a predominance of monoterpene and sesquiterpene hydrocarbons, with variation observed among elevation groups.

One-way ANOVA revealed that several volatile compounds exhibited statistically significant differences among elevation groups. In particular, β-pinene, β-myrcene, limonene, germacrene-D, and caryophyllene showed significant variation across elevations (*p* < 0.05). In contrast, some compounds did not exhibit significant differences among groups, indicating a relatively stable distribution across the studied elevations. These results indicate that elevation is associated with variation in the relative abundance of selected volatile compounds.

**Table 1:** Relative abundance (%) of volatile components identified in *Cedrus libani* leaf samples collected from three elevation levels (CLA: 1300 m, CLB: 1500 m, CLC: 1700 m).

					Relative Content (%)		
No	Components	Code	RIL	RIE	CLA	CLB	CLC
Monoterpene							
1	α-pinene	apine	932^27^	929	7.38 ± 0.59a	7.31 ± 0.31a	7.88 ± 1.01a
2	β-pinene	bpine	985^28^	976	0.79 ± 0.01b	0.66 ± 0.05b	4.29 ± 0.98a
3	β-myrcene	bmyrc	993^29^	989	17.02 ± 1.01b	19.28 ± 1.12a	10.61 ± 1.97c
4	o-cymene	ocyme	1028^30^	1021	0.41 ± 0.01a	0.33 ± 0.02b	0.32 ± 0.04b
5	α-terpinene	ater2	1016^29^	1013	0.15 ± 0.01b	0.19 ± 0.01b	0.25 ± 0.04a
6	cis-ocimene	cocim	1035^27^	1034	0.20 ± 0.01a	0.10 ± 0.00b	0.09 ± 0.00b
7	Camphene	camph	946^28^	943	0.10 ± 0.00b	0.16 ± 0.01a	0.14 ± 0.03a
8	Sabinene	sabin	969^28^	970	ND	0.22 ± 0.01a	0.12 ± 0.01b
9	l-phellandrene	lphel	1008^27^	1002	0.28 ± 0.01b	0.15 ± 0.00c	0.56 ± 0.03a
10	Δ³-carene	d3car	1008^31^	1004	0.09 ± 0.00c	0.30 ± 0.02a	0.25 ± 0.01b
11	Limonene	limon	1024^27^	1027	17.58 ± 2.05a	2.35 ± 1.52c	4.88 ± 1.95b
12	γ-terpinene	gterp	1054^27^	1054	0.11 ± 0.00c	0.21 ± 0.01a	0.16 ± 0.00b
13	α-terpinolene	aterp	1088^31^	1082	1.01 ± 0.06b	1.21 ± 0.05a	1.08 ± 0.01b
**Sesquiterpenes**							
14	α-cubebene	acube	1345^32^	1343	0.69 ± 0.01b	0.95 ± 0.02a	0.91 ± 0.11a
15	Cyclosativene	cyclo	1367^33^	1365	0.44 ± 0.10ab	0.47 ± 0.25a	0.45 ± 0.40a
16	α-copaene	acopa	1374^27^	1372	2.68 ± 0.12a	3.32 ± 1.12a	1.74 ± 1.01b
17	β-bourbonene	bbour	1387^27^	1379	1.12 ± 0.56a	1.04 ± 0.98a	1.19 ± 0.78a
18	Caryophyllene	caryo	1684^30^	1416	6.38 ± 1.12b	9.78 ± 1.93a	7.10 ± 1.99b
19	β-cubebene	bcube	1387^28^	1425	0.56 ± 0.05a	0.73 ± 0.21a	1.03 ± 0.86a
20	β-cadinene	bcadi	1462^35^	1441	0.91 ± 0.01a	1.16 ± 0.56a	1.27 ± 1.01a
21	cis-muurola-3,5-diene	cmuur	1452^27^	1444	0.54 ± 0.02b	0.68 ± 0.20ab	0.73 ± 0.11a
22	α-humulene	ahumu	1452^27^	1451	1.50 ± 0.66a	2.23 ± 1.01a	1.66 ± 1.01a
23	(+)-Epi-bicyclosesquiphellandrene	ebicy	1498^27^	1458	1.02 ± 0.61a	1.46 ± 0.95a	1.62 ± 0.75a
24	Cadina-1(6),4-diene < 10βH->	cadin1	1520^27^	1468	0.96 ± 0.21a	1.00 ± 0.95a	0.99 ± 0.66a
25	γ-muurolene	gmuur	1478^28^	1472	3.50 ± 1.01a	3.90 ± 1.14a	4.10 ± 1.57a
26	Germacrene-D	germa	1484^27^	1478	6.92 ± 1.22b	11.20 ± 2.05a	11.98 ± 2.56a
27	α-amorphene	aamor	1485^31^	1488	2.44 ± 1.05a	2.96 ± 2.12a	3.12 ± 1.15a
28	Epizonarene	epizo	1500^27^	1492	1.69 ± 0.66a	1.71 ± 0.80a	1.64 ± 0.61a
29	α-muurolene	amuur	1501^30^	1515	2.44 ± 0.82a	2.63 ± 1.15a	2.84 ± 1.21a
30	Δ-Cadinene	cadin	1527^27^	1499	0.90 ± 0.05a	1.04 ± 0.41a	1.08 ± 0.80a
31	γ-cadinene	gcadi	1515^30^	1509	3.87 ± 0.95a	4.47 ± 1.43a	5.01 ± 1.55a
32	δ-cadinene	dcadi	1522^27^	1515	6.55 ± 1.01a	6.85 ± 2.08a	7.19 ± 2.01a
33	Calamenene	calam	1508^27^	1518	1.07 ± 1.05a	0.56 ± 0.15a	0.90 ± 0.15a
34	Cadina-1,4-diene	cadin2	1510^27^	1528	0.81 ± 0.05b	0.97 ± 0.21ab	1.05 ± 0.20a
35	α-muurolene-(-)	amuu2	1502^27^	1532	1.73 ± 0.40a	2.00 ± 1.06a	2.25 ± 1.15a
36	α-calacorene	acalac	1535^27^	1536	0.78 ± 0.06b	0.60 ± 0.08b	1.95 ± 0.95a
37	β-elemene	belem	1389^30^	1385	0.16 ± 0.01c	0.31 ± 0.02a	0.28 ± 0.01b
**Terpenoids**							
38	Penten-3-one	pente	676^31^	686	0.16 ± 0.05a	0.12 ± 0.03ab	0.07 ± 0.01b
39	Hexanal	hexaa	799^31^	800	0.11 ± 0.01a	0.22 ± 0.01b	0.25 ± 0.02a
40	(E)-2-hexenal	e2hex	855^27^	847	2.56 ± 1.01b	3.14 ± 1.98ab	4.76 ± 1.56a
41	(Z)-hex-3-en-1-ol	zhex3	850^27^	851	1.62 ± 0.40a	1.00 ± 0.25b	1.33 ± 0.50ab
42	1-hexanol	hexao	865^27^	865	0.12 ± 0.04a	0.08 ± 0.01ab	0.05 ± 0.02b
43	tau-cadinol	tcadi	1620^34^	1651	0.23 ± 0.10a	0.25 ± 0.15a	0.35 ± 0.14a
44	2,8,8-Trimethyl-4-methylene-2- vinylbicyclo[5,2,0] nonane	trime	1418^33^	1330	0.29 ± 0.08a	0.36 ± 0.05a	0.34 ± 0.05a
45	Thymyl methyl ether	thyml	1215^36^	1226	0.11 ± 0.08b	0.29 ± 0.04a	0.16 ± 0.01b

RIL: retention index (literature); values in parentheses indicate literature references. RIE: experimentally determined retention index. ND: not detected. Values are expressed as mean ± standard deviation (SD). Different letters indicate statistically significant differences (*p* < 0.05).

**Fig. 1:**
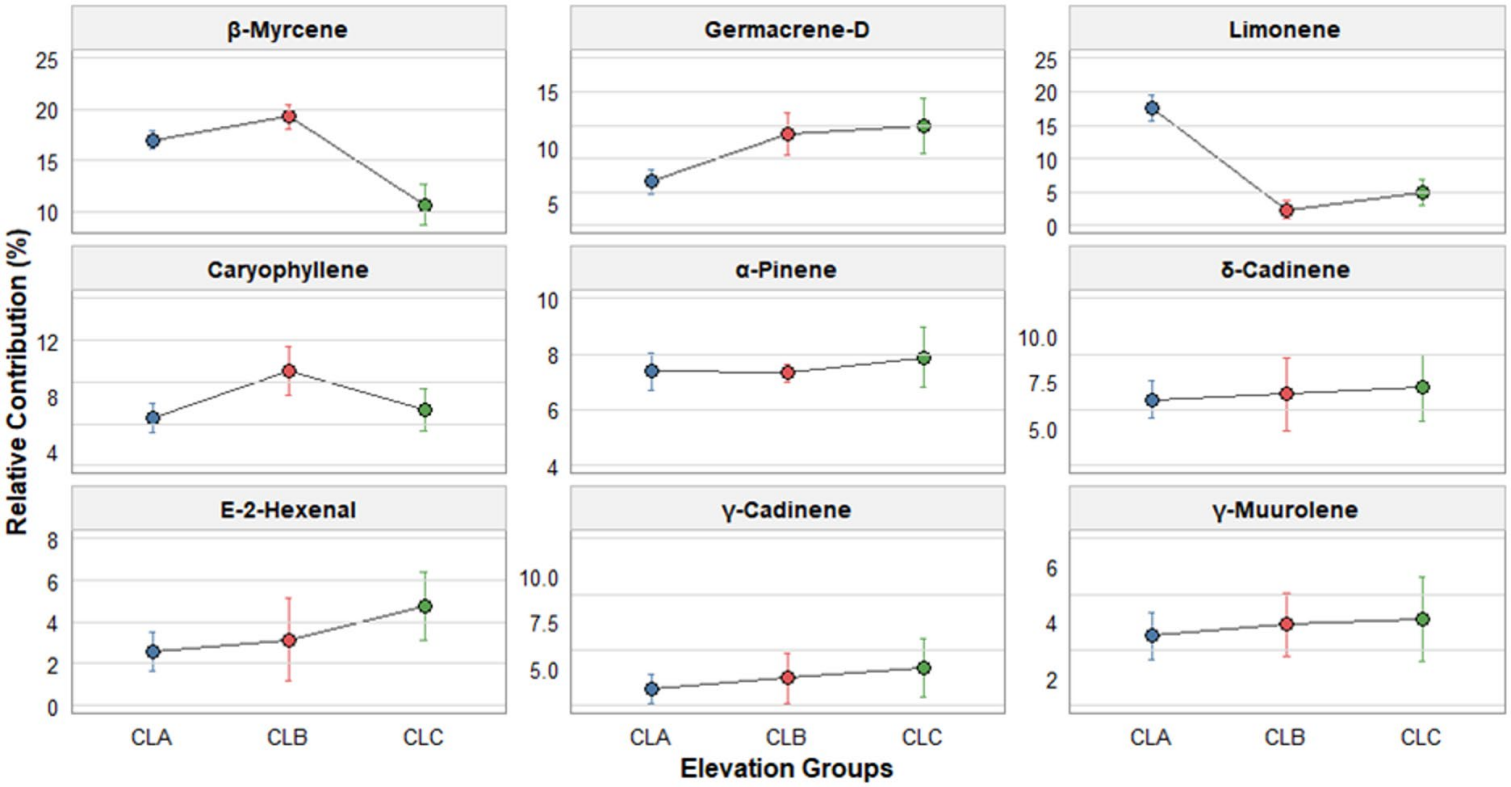
Variation in the relative contribution (%) of selected major volatile compounds (β-myrcene, germacrene-D, limonene, caryophyllene, α-pinene, δ-cadinene, (E)-2-hexenal, γ-cadinene, and γ-muurolene) across elevation groups (CLA, CLB, and CLC). Values are presented as mean ± standard deviation (SD).

Figure 1 presents the variation in the relative contribution (%) of selected volatile compounds detected in *C. libani* leaf samples collected from three elevation levels (CLA, CLB, and CLC). Each panel illustrates the relative abundance of individual compounds across elevation groups. Overall, variation in compound composition was observed among elevations, with different compounds exhibiting distinct distribution patterns.

### Content of flavonoid compounds

Among the flavonoid compounds identified in *C. libani* leaves, rutin and kaempferol showed decreasing trends with increasing elevation, whereas quercetin and catechin exhibited increasing trends (Table 2). Epicatechin content was lowest at mid-elevation. One-way ANOVA indicated that all analyzed flavonoid compounds showed statistically significant differences among elevation groups (*p* < 0.05), confirming the observed variation in flavonoid composition.

**Table 2:** Quantitative flavonoid composition (µg/g dry weight) of *Cedrus libani* leaves across elevation levels.

Flavonoid compounds (µg/g)	CLA	CLB	CLC
Flavonols			
Rutin	66.7 ± 0.001a	34.6 ± 0.013b	21.3 ± 0.153c
Quercetin	53.7 ± 0.014c	69.7 ± 0.017b	95.1 ± 0.029a
Kaempferol	14.2 ± 0.004a	13.7 ± 0.017b	13.4 ± 0.024c
**Flavan-3-ols**			
Epicatechin	54.7 ± 0.004b	42.7 ± 0.027c	76.5 ± 0.015a
Catechin	417.0 ± 1.225c	673.1 ± 0.971a	535.6 ± 1.351b

Values are expressed as mean (µg/g dry weight) ± SD. Different letters indicate statistically significant differences (*p* < 0.05).

**Fig. 2:**
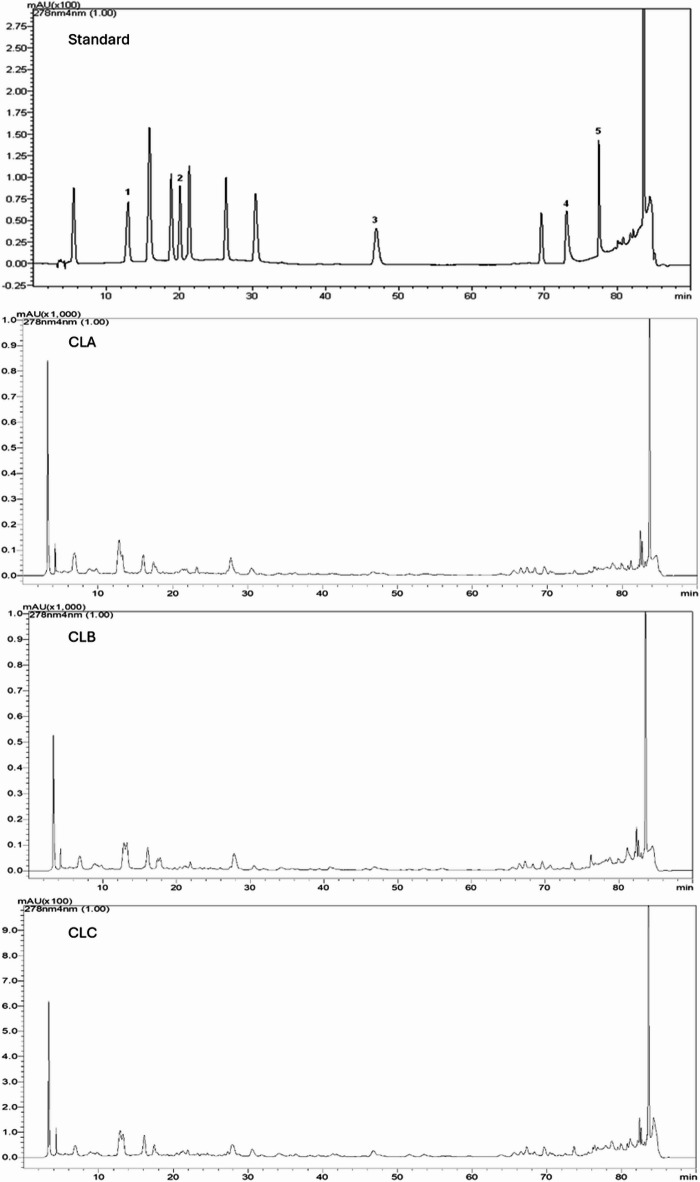
HPLC–DAD chromatogram of flavonoid standards recorded at 278 nm. Peaks correspond to (1) catechin, (2) epicatechin, (3) rutin, (4) quercetin, and (5) kaempferol. Compound identification was based on retention time matching with authentic standards.

In the standard chromatogram recorded at 278 nm, flavonoid compounds were observed as distinct peaks within the analyzed retention time range (Fig. 2). Based on these retention times, flavonoid compounds in leaf extracts collected from different elevations (CLA, CLB, and CLC) were identified, and quantitative analyses were performed. Differences in flavonoid composition were observed among elevation groups.

### Principal component analysis (PCA)

**Fig. 3:**
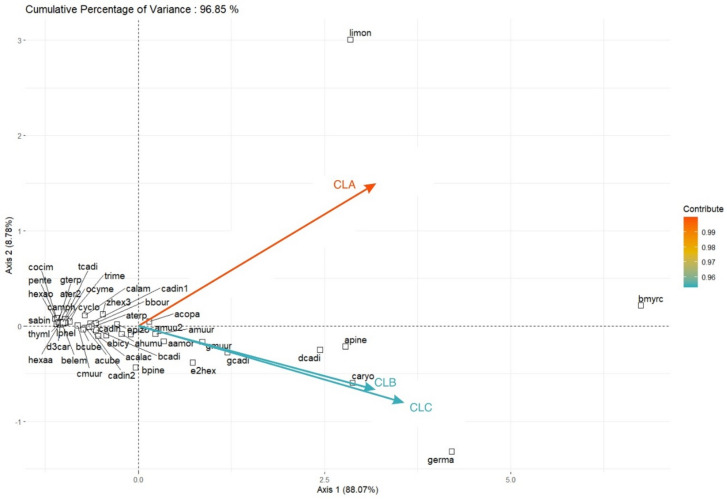
PCA biplot showing the distribution of samples (CLA, CLB, and CLC) and volatile organic compounds (VOCs) based on chromatography-derived relative abundance (%) data. The analysis was performed using normalized data to evaluate compositional differences among elevation levels.

PCA was applied to explore patterns of variation in volatile compound composition among samples (Fig. 3). The first two principal components accounted for the majority of the total variance in the dataset. The biplot shows a separation of samples (CLA, CLB, and CLC) along the principal component axes, reflecting differences in their relative compound composition.

Several compounds, including limonene, β-myrcene, α-pinene, caryophyllene, and germacrene-D, contributed to the observed distribution pattern. Samples were positioned differently in the multivariate space, indicating variation in compositional profiles among elevation groups. However, given the limited number of elevation levels, these patterns should be interpreted cautiously as descriptive. The high variance explained by the first principal component likely reflects data structure and collinearity rather than necessarily a biologically meaningful gradient.

**Fig. 4:**
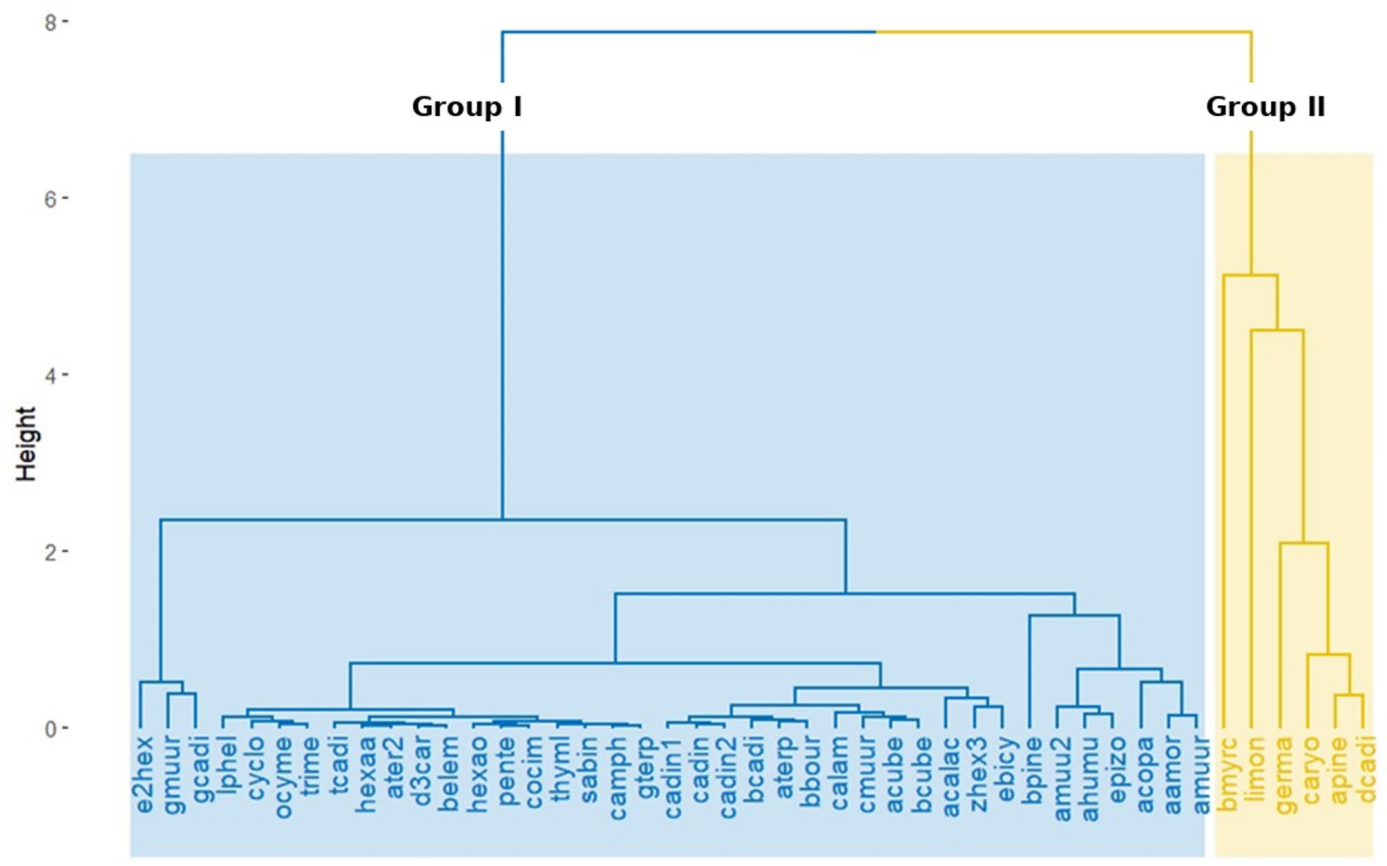
Hierarchical cluster analysis (HCA) dendrogram of volatile organic compounds based on chromatography-derived relative abundance (%) data. Two main groups (Group I and Group II) were identified.

Cluster analysis revealed two main groups in the dendrogram (Fig. 4). The larger group (Group I) included a high number of compounds showing similarity in their compositional patterns, whereas the smaller group (Group II) was separated at a higher linkage distance. This separation indicates differences in compound distribution patterns, with compounds such as β-myrcene, limonene, germacrene-D, caryophyllene, α-pinene, and δ-cadinene contributing to this grouping.

## Discussion

Previous studies on *C. libani* have reported the identification of up to 56 volatile constituents^37^, whereas 52 components were detected in the present study. However, only 45 compounds were included in the comparative analysis, as several minor constituents were detected at trace levels or were not consistently present across all elevations. Among the shared components, camphene exhibited notable quantitative differences: whereas the previous study reported a content of 2.4%, in the present study it ranged between 0.10% and 0.16%. Such variations may be attributed to differences in geographical origin, climatic conditions, the developmental stage of the plant, or extraction methodology. In a study conducted on Norway spruce, altitude was reported to influence monoterpenes and sesquiterpenes, with the content of all terpenes decreasing as elevation increased^13^. Furthermore, the same study emphasized that terpene biosynthesis, which has been reported in the literature to be associated with stress-related processes in evergreen coniferous trees, is closely linked to growth conditions. The observed differences in the present study may therefore be associated with these elevation-dependent environmental influences.

In a study conducted on *C. libani* specimens collected from different regions of Lebanon, it was reported that extracts obtained from the leaves had a lower overall yield compared to those from the cones, yet exhibited a higher diversity of components^21^. In their findings, germacrene-D (29.40%) was identified as the major component. Similarly, germacrene-D was also identified among the predominant compounds, ranging between 6.92% and 11.98%. Moreover, the proportions of δ-cadinene (5.60%), γ-muurolene (4.83%), γ-cadinene (2.87%), and β-pinene (0.54%) reported in the same study were comparable to those observed in the present study. However, the contents of α-pinene and β-myrcene were found to be higher in the present study. In addition, the flavonoid composition of the samples was analyzed, revealing the presence of rutin (154.33 µg/g), quercetin (2 µg/g), kaempferol (5 µg/g), and resveratrol (20 µg/g). When compared with the present study, the content of rutin was found to be lower in that study, whereas the levels of quercetin and kaempferol were higher.

A study was conducted to examine the regional variations in yield and chemical composition obtained from the cones of *C. libani* individuals growing at different altitudes and geographical regions in Lebanon^38^. The main volatile constituents identified were α-pinene (25.1–37.3%), β-pinene (6.4–35.6%), myrcene (0-30.6%), and limonene (5.6–14.1%). Similarly, in the present study, these compounds were also determined to be among the predominant constituents. According to the PCA results of that study^38^, PC1 (72%) represented α-pinene and β-pinene, and limonene derivatives, whereas PC2 (10.9%) reflected the variation in myrcene content. The Qartaba population was distinctly separated from the others due to its high myrcene concentration. In the present study, comparable separation among samples was also observed, with similar compounds contributing to the variation along principal component axes. Both studies emphasized that environmental conditions, particularly altitudinal and geographical differences, may influence the quantitative and qualitative composition. The findings of the present study are therefore consistent with those of that study, indicating that elevation may contribute to variation in volatile components in *C. libani*. Similarly, it has been reported that the limonene content in *C. deodora* was 17.01%^20^. In the present study, the *C. libani* samples collected from the lowest altitude (CLA) showed a comparable but slightly higher limonene content of 17.58%. In the same study, the caryophyllene content was 3.14%, whereas in our samples it ranged from 6.38% to 9.78%, indicating possible interspecific and environmental variation in sesquiterpene profiles. It has been reported that δ-cadinene accounted for 3.65% of *C. atlantica*^39^. In our *C. libani* samples, δ-cadinene was found at higher levels, ranging from 6.55% to 7.19%. This difference may reflect variation among studies and environmental conditions, including those associated with elevation. PCA results indicated separation among samples based on chemical composition. However, given the limited number of elevation levels, these patterns should be interpreted cautiously as descriptive. The high variance explained by PC1 likely reflects the underlying data structure and collinearity among variables, rather than a clearly defined biological gradient.

In a previous study conducted on *Pinus nigra* subsp. *pallasiana* and *C. libani*, the major components were identified as α-pinene, β-pinene, limonene, β-caryophyllene, and germacrene-D, and HPLC analysis revealed the presence of rutin, quercetin, and kaempferol in varying proportions^24^. In the present study, germacrene-D was likewise among the predominant compounds; however, its proportion (6.92–11.98%) was lower compared to values reported in some earlier studies. While α-pinene and β-pinene were detected in our samples, they were not consistently the dominant constituents across elevations, indicating possible altitude-related compositional variation. Similarly, study^25^ reported that α-pinene and β-pinene were the major components of seed in three *C. libani* populations from the Isparta region and identified significant differences (*p* < 0.05) among and within populations with respect to age, altitude, and diameter at breast height classes. Our findings are consistent with these observations, as clear quantitative differences were detected among elevations, indicating variation associated with altitude and related ecological factors. Regarding phenolic compounds, study^24^ reported the presence of rutin, quercetin, and kaempferol in different proportions between species. In the present study, all three flavonols were likewise detected; however, distinct elevation-dependent trends were observed. Rutin content decreased with increasing altitude (66.7–21.3 µg/g), whereas quercetin increased markedly (53.7–95.1 µg/g), and kaempferol remained relatively stable. These differences further indicate that environmental gradients, particularly elevation, may be related to variation in both volatile and flavonoid profiles in *C. libani*.

In the present study, distinct variation patterns in volatile compounds were observed across elevations. β-myrcene reached its highest relative abundance at medium elevation, while limonene showed higher levels at low altitude with a decreasing trend toward higher elevations. Similarly, β-caryophyllene exhibited its maximum values at medium elevation, whereas compounds such as α-pinene and δ-cadinene showed a slight increase with increasing altitude. β-pinene also displayed higher levels in samples collected at higher elevations. In contrast, (Z)-3-hexenol remained relatively stable across all elevations.

Overall, the volatile compound profile varied across elevations, with certain compounds showing distinct distribution patterns along the altitudinal gradient. Moderate elevation (CLB) was associated with relatively higher levels of several compounds, whereas some compounds showed increased relative abundance at higher elevations (CLC). These patterns indicate that volatile compound composition in *C. libani* varies along the altitudinal gradient. However, in the absence of direct environmental or physiological measurements, these differences should be interpreted as patterns of variation rather than evidence of specific functional or adaptive mechanisms.

Elevation in this study should be considered as a proxy for multiple co-varying environmental factors. Altitudinal variation in secondary metabolite profiles of higher plants has not yet been comprehensively investigated, and the number of available studies remains relatively limited. Most existing studies have been conducted on plants growing in their natural habitats, where the contribution of genetic differences between low-altitude and high-altitude populations to the observed phytochemical variation cannot be entirely ruled out^40^. As elevation increases, numerous environmental parameters change simultaneously, including precipitation, mean temperature, daily thermal amplitude, soil fertility, wind speed, atmospheric pressure, duration of snow cover, length of the vegetation period, and radiation intensity^41^. In line with previous reports, the findings of the present study indicate that both volatile components and flavonoid compounds in *C. libani* vary along the altitudinal gradient. The observed shifts in terpene composition and flavonoid content may be related to elevation-related environmental variation. Given that multiple abiotic factors co-vary with altitude, the detected differences in metabolite profiles may reflect a combined response to environmental variation rather than the effect of a single factor. Moreover, the concurrent variation observed in both volatile terpenoids and flavonoid compounds may be related to altitude-related environmental conditions, particularly decreasing temperature and changes in radiation regimes, which may contribute to variation in the chemical profile of *C. libani.*

This study has several limitations that should be considered when interpreting the results. First, the number of biological replicates is limited, as samples at each elevation level were collected from a restricted number of trees, which may not fully represent intraspecific variation. Second, only three elevation levels within a single forest area were examined; therefore, the observed patterns may be confounded with other environmental factors such as soil properties, microclimate, and stand structure. Third, environmental variables (e.g., temperature, UV radiation) were not directly measured, limiting the ability to attribute the observed chemical variation to specific environmental drivers. In addition, the flavonoid analysis was based on a targeted approach focusing on a limited number of compounds, and other phenolic constituents were not characterized. Therefore, the findings of this study should be interpreted as preliminary and descriptive rather than definitive or mechanistic.

## Materials and methods

### Sampling of plant material

**Fig. 7:**
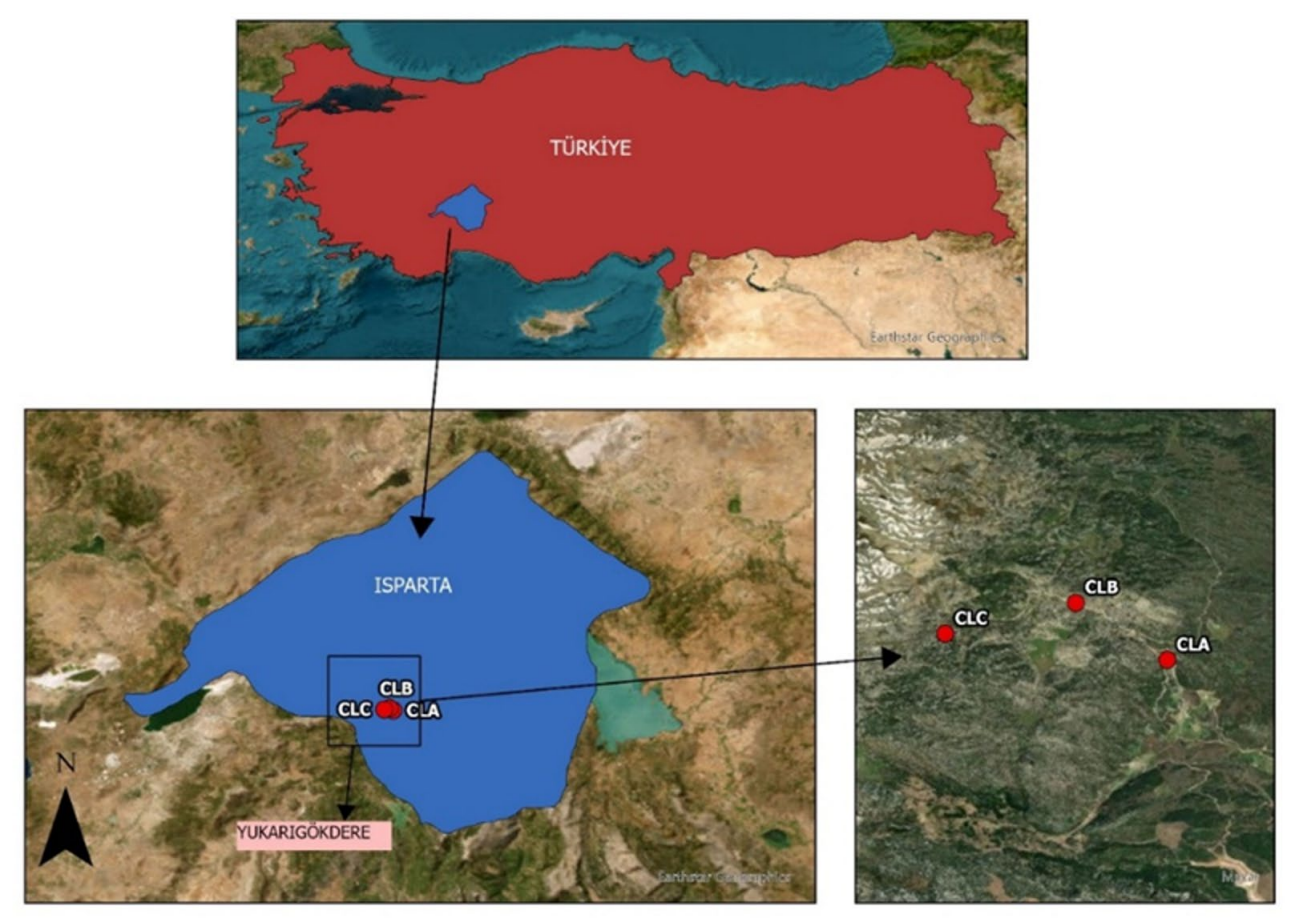
Map of the study area showing sampling locations across three elevation levels (CLA, CLB, CLC). The map was produced by the author using ArcGIS Pro version 3.1.

This study was conducted within the borders of the Yukarı Gökdere Forest Management Directorate in the Lake District of Turkey. Spatial visualization of the sampling locations was performed using ArcGIS Pro (version 3.1; Esri Inc., Redlands, CA, USA) (Fig. 7). The basemap layers used in the map were provided by Esri. Leaves from *C. libani* trees approximately 75 years old and growing naturally in the region were collected in May 2025 at three elevations: 1300 m (CLA), 1500 m (CLB), and 1700 m (CLC) (Table 3). At each elevation, seven independent and mature trees were randomly selected, and each tree was treated as a biological replicate. Freshly collected leaves were transported in containers insulated with ice packs and stored at 4 °C until analysis. Leaves were randomly selected from healthy and fully developed tissues and were collected consistently from the east-facing side of the canopy to minimize the effects of exposure variability. Special care was taken to ensure that all sampled trees were of similar age. Sampling at all elevations was conducted on the same day under comparable weather conditions, thereby reducing the potential influence of temporal and climatic variability on the results. *C. libani* is listed as a vulnerable species (VU) according to the IUCN Red List of Threatened Species. The collection of plant material complied with all relevant institutional, national, and international guidelines and legislation, including the IUCN Policy Statement on Research Involving Species at Risk of Extinction and the Convention on International Trade in Endangered Species of Wild Fauna and Flora (CITES).

**Table 3:** Sampling information and geographical coordinates of leaf samples.

Sample	Location	Elevation	Aspect	Age	Coordinates (UTM)
CLA	Yukarı Gökdere	1300 m	East	75	308,743–4,177,035
CLB	Yukarı Gökdere	1500 m	East	75	307,748–4,177,851
CLC	Yukarı Gökdere	1700 m	East	75	306,317–4,177,459

### Plant material identification

The plant material was taxonomically identified by Prof. Dr. Hasan OZCELİK, Department of Botany, Süleyman Demirel University, Turkey. A voucher specimen (Voucher No. GUL-PI-3/1–3) has been deposited in the GUL Herbarium of Süleyman Demirel University (GULH), Isparta, Turkey.

### Determination of volatile compounds (HS-SPME/GC–MS)

The technique described by^42^ was used to determine the compositions of the leaves. After precisely weighing 5 g of the sample, it was placed into a 15 mL glass vial sealed with a silicone septum. The vial was then incubated for 15 min to ensure thermal equilibration at 45 °C using a heating block. To aid in the adsorption of volatile components present in the leaf matrix, a Carboxen/PDMS solid-phase microextraction (SPME) fiber (75 μm fused silica, Supelco Ltd., Bellefonte, PA, USA) was manually inserted into the vial headspace and maintained for 30 min at 45 °C. The CAR/PDMS fiber was selected due to its high affinity for low-molecular-weight volatile compounds, particularly monoterpenes commonly reported in conifer species. The extraction temperature and duration were chosen based on previously reported studies and established analytical conditions in the literature. To allow thermal desorption of the adsorbed volatile compounds, the fiber was then inserted into the injection port of the gas chromatograph and maintained at 250 °C for five minutes. Leaf samples were collected from seven independent trees at each elevation level, and each tree was treated as a biological replicate. Each sample was analyzed under identical experimental conditions.

A Shimadzu GC-2010 system and an MS-QP2010 Plus mass spectrometer (Shimadzu Corporation, Kyoto, Japan) were used for the gas chromatography-mass spectrometry (GC-MS) analysis. An Rxi-5Sil MS capillary column (30 m length × 0.25 mm inner diameter × 0.25 μm film thickness; Restek, Bellefonte, PA, USA) was used to accomplish the analytical separation. At a rate of 4 °C per minute, the oven temperature was programmed to rise from 40 °C (held for two minutes) to 250 °C (held for five minutes). An inlet pressure of 83.5 kPa and an injector temperature of 250 °C were established. Helium (purity ≥ 99.999%, Linde, Germany) was used as the carrier gas at a linear velocity of 44.2 cm/s. Split mode (10:1) was used for the injection. The interface temperature of the mass spectrometer was kept at 250 °C, and the ionization technique used was electron ionization (EI). The mass spectrometer functioned at a scan speed of 1428 u/s, an acquisition interval of 0.30 s (2 Hz), and a detector voltage of 1.5 kV over a mass scan range of 35–450 m/z. Shimadzu GCMSsolution software (version 2.5) was used for data collection and processing. Volatile compounds were expressed as relative abundance (%), calculated by normalizing individual peak areas to the total chromatographic area of each sample.

By comparing their mass spectra and retention indices (RIs) with those of genuine standards (α-pinene, β-pinene, β-myrcene, o-cymene, α-terpinene, cis-ocimene, camphene, sabinene, L-phellandrene, Δ³-carene, limonene, γ-terpinene, α-terpinolene, α-cubebene, cyclosativene, α-copaene, β-bourbonene, caryophyllene, β-cubebene, β-cadinene, cis-muurola-3,5-diene, α-humulene, (+)-epi-bicyclosesquiphellandrene, cadina-1(6),4-diene (< 10βH> ), γ-muurolene, germacrene-D, α-amorphene, epizonarene, α-muurolene, cadinene, γ-cadinene, δ-cadinene, calamenene, cadina-1,4-diene, α-muurolene (–), α-calacorene, β-elemene, penten-3-one, hexanal, (E)-2-hexenal, (Z)-hex-3-en-1-ol, hexanol, τ-cadinol, 2,8,8-trimethyl-4-methylene-2-vinylbicyclo[5.2.0]nonane, and thymyl methyl ether), all obtained from Sigma-Aldrich (Merck, Germany), the volatile components were identified. Further confirmation was obtained using spectral databases (NIST MS ‘23 and Wiley Flavors/Fragrances (FFNSC)). A homologous series of n-alkanes (C7–C30; Sigma-Aldrich, Germany) was used to calculate retention indices.

### Determination of flavonoid compounds (Extraction and RP-HPLC–DAD)

5 g of dried and ground leaf samples were extracted using 100 mL of a solvent mixture consisting of acetonitrile, methanol, and acetic acid (50:49.5:0.5, v/v/v). Authentic standards (purity ≥ 95–99%) were obtained from Sigma-Aldrich (Merck, Germany). After homogenization, the mixture was subjected to ultrasonic extraction for 2 h in a bath sonicator (Super RK 255 H, Bandelin electronic, Berlin, Germany). The resulting extract was filtered through Whatman No. 1 filter paper using a Büchner funnel. The filtrate was then concentrated to a constant weight under reduced pressure (Rotavapor R-210, Büchi, Switzerland; T < 40 °C) and subsequently redissolved in methanol. The flavonoid composition of *C. libani* leaves was analyzed using reversed-phase high-performance liquid chromatography (RP-HPLC). The HPLC system consisted of an SCL-10Avp system controller, SIL-10ADvp autosampler, LC-10ADvp pump, DGU-14 A degasser, CTO-10Avp column oven, and a diode-array detector (DAD; SPD-M20A, Shimadzu Corporation, Kyoto, Japan), with detection performed at 278 nm^43^. Chromatographic separation was achieved using an Agilent Eclipse XDB-C18 column (250 mm × 4.6 mm i.d., 5 μm). The column temperature was maintained at 30 °C, the mobile phase flow rate was set at 0.8 mL/min, and the injection volume was 20 µL. Gradient elution was carried out according to the program shown in Table 4, using solvent A (acetic acid–water, 3:97, v/v) and solvent B (methanol).

**Table 4:** Solvent gradient conditions with linear gradient.

Final time (min)	A (%)	B (%)	Final time (min)	A (%)	B (%)
0.10	93	7	62	58	42
20	72	28	70	50	50
28	75	25	73	30	70
35	70	30	75	20	80
50	70	30	80	0	100
60	67	33	81	93	7

Chromatographic data were processed and quantified using Shimadzu Class-VP Chromatography Laboratory Software. All leaf extracts, standard solutions, and mobile phases were filtered through 0.45 μm pore-size membrane filters prior to analysis. Quantification of flavonoid compounds was performed using external calibration curves constructed for each authentic reference standard. The determination of flavonoid profiles followed a modified version of the method described by^44^. All determinations were performed in triplicate using three independent samples. The flavonoid compounds, rutin, quercetin, kaempferol, epicatechin, and catechin, identified and quantified using authentic standards obtained from Sigma-Aldrich (Merck, Germany), were analyzed in the leaves of *C. libani* A. Rich. These compounds were selected based on previous studies on the *Cedrus* genus and *C. libani*, which reported their high abundance and emphasized their biological and pharmacological significance^21^.

Quantification of flavonoid compounds (catechin, epicatechin, rutin, quercetin, and kaempferol) was performed using external standard calibration curves (Table 5). Calibration equations were established in the form of *Y = A + BX*, where *Y* represents peak area and *X* represents concentration. All calibration curves showed high linearity, with correlation coefficients (R²) ranging from 0.9977 to 0.9999. The limits of detection (LOD) and limits of quantification (LOQ) were determined for each compound. LOD values ranged from 0.03 to 0.10 µg/g, while LOQ values ranged from 0.08 to 0.40 µg/g.

**Table 5:** Calibration parameters of flavonoid standards.

Compound	Equation (Y = A+BX)	*R*²	LOD (µg/g)	LOQ (µg/g)
Catechin	Y = 25173.5 + 16519.4X	0.9998	0.10	0.30
Epicatechin	Y = 11443.5 + 19310.5X	0.9999	0.10	0.40
Rutin	Y = 5453.3 + 21424.8X	0.9999	0.05	0.15
Quercetin	Y = -24713.0 + 49887.1X	0.9993	0.06	0.19
Kaempferol	Y = 49393.3 + 80859.7X	0.9977	0.03	0.08

### Statistical analysis

All data were expressed as mean ± standard deviation (SD) based on biological replicates. Prior to analysis, the normality of the data distribution and homogeneity of variances were assessed. Differences among elevation groups were evaluated using one-way analysis of variance (ANOVA), followed by Tukey’s honestly significant difference (HSD) post-hoc test for multiple comparisons. Statistical significance was accepted at *p* < 0.05, and different letters were used to indicate statistically significant differences between groups. All statistical analyses were performed using SPSS software (version 16.0; SPSS Inc., Chicago, IL, USA).

### PCA and HCA

Results are presented as mean ± SD based on seven biological replicates per elevation level. Principal component analysis (PCA) was applied to investigate multivariate relationships among dominant components (≥ 5%) and quantified flavonoid compounds across elevations. Data were mean-centered and scaled prior to analysis. PCA and visualization were conducted in RStudio using the FactoMineR and ggplot2 packages^45–47^. Hierarchical cluster analysis (HCA) was performed using Euclidean distance and Ward’s method to assess similarity patterns among samples.

Multivariate analyses (PCA and HCA) were performed using normalized relative abundance (%) data. This approach allows comparison of compositional patterns among samples; however, it does not represent absolute quantitative differences or concentrations. Therefore, these analyses should be interpreted as exploratory and descriptive, reflecting relative variation in compound composition rather than confirmatory statistical evidence.

## Conclusion

In this study, variation in the phytochemical composition of *C. libani* leaves was observed across different elevation levels. A total of 45 volatile compounds were identified, predominantly consisting of monoterpenes and sesquiterpenes, with key components such as limonene, β-myrcene, α-pinene, caryophyllene, and germacrene-D showing variation among elevation groups. Flavonoid profiles also varied, with rutin and kaempferol decreasing, while quercetin and catechin showed relatively higher values at higher elevations.

Multivariate analyses (PCA and HCA) revealed compositional differences among samples; however, these patterns should be interpreted as descriptive due to the limited sampling design.

Overall, the findings indicate that phytochemical composition in *C. libani* leaves varies along the studied elevation gradient. This study provides a preliminary characterization of altitude-related chemical variation and highlights the need for future studies incorporating broader spatial sampling and direct environmental measurements to better understand the drivers of these patterns.

## Data Availability

All data supporting the findings of this study are available within the paper.
